# Strain-Specific Impact of Titanium Dioxide Nanoparticles on *Fremyella Diplosiphon* Physiological and Metabolic Responses

**DOI:** 10.1007/s00284-025-04629-9

**Published:** 2025-12-01

**Authors:** Mst Sayadujjhara, Yavuz S. Yalcin, William Ghann, Jamal Uddin, Viji Sitther

**Affiliations:** 1 Department of Biology, Morgan State University, Baltimore, MD 21251 USA; 2 Center for Nanotechnology, Department of Natural Sciences, Coppin State University, 2500 West North Avenue, Baltimore, MD 21216 USA

**Keywords:** Adenosine triphosphate, Photosynthesis, Pigment autofluorescence, Reactive oxygen species, Titanium dioxide nanoparticles

## Abstract

Titanium dioxide nanoparticles (n-TiO₂) have emerged as potent modulators of photosynthetic activity in cyanobacteria; however, their strain-specific physiological effects in *Fremyella diplosiphon*, a model cyanobacterium, remain unexplored. In this study, we investigated the impact of n-TiO_2_ on growth, pigment autofluorescence, photosynthetic capacity, reactive oxygen species (ROS) generation and ATP synthase activity in *F. diplosiphon* strains B481-SD (overexpressed with the sterol desaturase gene) and B481-WT (wild type). Growth as a measure of optical density was maximal in B481-SD at 2.0 mg/L (0.67 ± 0.01) and in B481-WT at both 2.0 (0.55 ± 0.01) and 16 mg/L (0.52 ± 0.01) n-TiO_2_ on day 12. Pigment accumulation over 15 days revealed enhanced phycocyanin (1300 ± 2) and chlorophyll *a* (890 ± 5) levels in 2.0 mg/L n-TiO₂-treated B481-SD while no significant changes were observed in B481-WT. Photosynthetic efficiency (*Fv/Fm*) of B481-SD treated with 2.0 mg/L n-TiO₂ was significantly higher on days 6, 9, and 12. ROS quantification using the 2′,7′dichlorodihydrofluorescein diacetate assay revealed significantly higher levels in B481-WT at 2.0 mg/L (260 ± 5), whereas B481-SD exhibited lower ROS levels at 2.0 (210 ± 2) and 16 mg/L (220 ± 4) n-TiO_2_ on day 15. Additionally, immunodetection analysis of ATP synthase revealed significantly enhanced expression in *F. diplosiphon* B481-SD treated with 0.5, 2.0, and 128 mg/L n-TiO₂ compared to the untreated control. Visualization of cell-n-TiO₂ interactions using field emission scanning electron microscopy equipped with energy-dispersive X-ray spectroscopy revealed a strong absorption for titanium, with an atomic percentage of 0.32%. These findings demonstrate strain-specific responses of *F. diplosiphon* to n-TiO₂, paving the way for scale-up cultivation to enhance cyanobacteria-derived bioproducts.

**Supplementary Information:**

The online version contains supplementary material available at 10.1007/s00284-025-04629-9.

## Introduction

Titanium dioxide (TiO₂) nanoparticles have garnered significant attention due to their distinct physicochemical properties and wide-ranging applications in environmental remediation, energy production, and biotechnology [1]. Recent studies have highlighted the impact of n-TiO₂ on cyanobacteria, which are key aquatic microorganisms involved in photosynthesis and nitrogen fixation [2, 3]. By modulating stress responses and altering metabolic activities, these nanoparticles can profoundly influence cyanobacterial physiology, thereby altering their ecological functions and interactions within aquatic ecosystems [4]. The interaction of n-TiO₂ with cyanobacterial photosynthetic machinery and cellular antioxidant systems offers a potential pathway to enhance metabolic resilience under environmental stress [5]. Understanding these interactions is critical for assessing the benefits and risks associated with n-TiO₂ exposure in natural ecosystems, as well as for harnessing their potential for biotechnological applications.

As primary producers in aquatic ecosystems, cyanobacteria contribute to approximately 20% of global oxygen production and efficiently capture light energy through specialized pigments such as phycobiliproteins and chlorophyll *a*, along with a highly active photosystem II (PSII) [6]. Studies on cyanobacteria such as *Synechococcus*, *Anabaena*, and *Microcystis* sp. have demonstrated that exposure to n-TiO₂ can significantly influence key cellular processes, including photosynthesis, growth, and metabolism, through both toxic and protective mechanisms [7, 8]. Interestingly, the effects of n-TiO₂ on these microorganisms can vary depending on the dose concentration, with low concentrations often promoting cellular growth and pigment accumulation, a phenomenon known as the compensatory effect [9]. Understanding the ability of optimal n-TiO₂ to enhance light capture, improve PSII efficiency, and optimize photosynthetic processes can significantly advance cyanobacteria-based biotechnological applications.

Adenosine triphosphate (ATP), the primary cellular energy currency, is synthesized by ATP synthase and plays a central role in almost every cellular metabolic process [10]. The introduction of n-TiO_2_ into cyanobacterial systems can significantly impact ATP production by altering light penetration and enhancing photosynthetic efficiency. A study by Cherchi et al. [3] demonstrated the upregulation of genes associated with cellular nitrogen uptake in *Anabaena* PCC 7120 at concentrations ranging from 6 to 60 mg/L/h n-TiO₂, supporting essential cellular functions such as energy transfer, metabolic regulation, and adaptation to environmental changes. However, at higher concentrations, n-TiO₂ can trigger excessive ROS production, leading to cellular damage. This oxidative impact has been shown to impair ATP synthesis and compromise overall cyanobacterial health in the nitrogen-fixing species *Anabaena* PCC 7120 [3]. As crucial molecules in cellular signaling, ROS are oxygen-derived molecules that play critical roles in cellular signaling pathways and homeostasis; however, excessive oxidative stress can lead to irreversible damage of cellular components, including proteins, lipids, and DNA [11]. While ROS generation is a natural byproduct of photosynthesis and cellular respiration in cyanobacteria, cellular mechanisms like non-photochemical quenching and increased antioxidant enzyme activity help protect these organisms from oxidative damage [10].

*F. diplosiphon* serves as a key model organism for studying cyanobacterial adaptation, particularly due to its unique light-regulated complementary chromatic mechanism [12]. Its rapid growth cycle (8–10 days) and ability to acclimate to diverse light conditions make it an ideal system for investigating cellular and metabolic dynamics. In this study, we investigated cell–nanoparticle interactions and the impact of n-TiO_2_ on key physiological and metabolic parameters in *F. diplosiphon* strains B481-SD (sterol desaturase-overexpressing strain, accession MH329183) and B481-WT (wild type, UTEX Algal Repository, Texas). These insights aim to enhance the potential of these strains for applications in biofuel production, carbon sequestration, pigment-derived nutraceuticals, and environmental remediation.

## Materials and Methods

### Strains and Culture Conditions

*F. diplosiphon* strains (B481-SD and B481-WT) were cultivated in liquid BG-11 medium supplemented with 20 mM HEPES. Cultures were grown in 250 mL flasks under constant agitation at 170 rpm and a temperature of 28 °C in an Innova 44R incubator shaker (Eppendorf, Hamburg, Germany). The light spectrum for photosynthesis was adjusted to 400–700 nm, with an irradiance of 30 µmol m^− 2^ s^− 1^. Titanium dioxide powder (Degussa P-25, 20 nm) was obtained from the Institute of Chemical Education, University of Wisconsin-Madison (Madison, WI, USA).

### Growth of *F. diplosiphon* B481-SD and B481-WT Strains in Varying n-TiO_2_ Concentrations

The effect of n-TiO_2_ on the growth of *F. diplosiphon* B481-SD and B-481WT across a range of concentrations (0.5, 1, 2, 4, 8, 16, 32, 64, and 128 mg/L) was measured using a UV-Vis (Agilent Technologies, CA, USA) spectrophotometer. Cellular suspensions were initially adjusted to an optical density of 0.1 at 750 nm (OD_750_) according to the procedure mentioned above. Measurements of OD_750_ were baseline-adjusted using the n-TiO_2_-only control for each concentration. The growth was measured at three-day intervals for a period of 15 days.

### Pigment Autofluorescence in n-TiO_2_ Treated *F. diplosiphon* B481-SD and B481-WT Strains

Phycocyanin and chlorophyll *a* autofluorescence in 0.5 to 128 mg/L n-TiO_2_-treated cultures and the untreated control were measured every three days using a BioTek Synergy H1 Microplate Reader (Agilent, United States) as described by Yalcin et al. [13]. A negative control of bare n-TiO_2_ was used to account for any potential interference in pigment autofluorescence. Specifically, chlorophyll autofluorescence was captured at an excitation wavelength of 420 nm and an emission wavelength of 680 nm, while phycocyanin fluorescence was assessed at an excitation wavelength of 590 nm with emissions at 650 nm. In addition, minimal (*Fo*) and maximal fluorescence yields (*Fm*) were measured at three-day intervals using a MINI-PAM (Walz, Germany), after a 15-minute dark incubation. The PSII quantum yield (*Fv/Fm*) was subsequently calculated based on the formula *Fv/Fo* = (*Fm - Fo*)/*Fo* as detailed by Wan et al. [14]. Three biological replicates were maintained for each strain and the experiment was repeated once.

### Detection of Intracellular ROS in n-TiO_2_-treated *F. diplosiphon* B481-SD and B481-WT Strains

Using the 2′,7′-dichlorodihydrofluorescein diacetate (DCFH-DA) fluorometric probe (EMD Chemicals, USA) [15], intracellular ROS levels in B481-SD and B481-WT strains were assessed in the untreated control, 0.5, 2.0, 16, and 128 mg/L n-TiO_2_, representing a gradient of low, moderate, and extreme cytotoxic effects. Cultures were grown for 15 days under conditions mentioned in Sect. 2.1, with three biological replicates for each treatment group, and the experiment was repeated once. For each strain, a negative control containing bare n-TiO₂ was included to account for potential confounding effects due to autofluorescence. A fresh 20 mM DCFDA stock was prepared and 50 µL added to 150 µL of the culture in a 96-well plate [16]. Fluorescence intensity was measured at an excitation of 495 nm and emission of 529 nm using the BioTek microplate reader after incubation in the dark for 45 min at room temperature.

### ATP Synthase Activity in n-TiO_2_ Treated *F. diplosiphon* Strains

#### Protein Extraction and SDS Polyacrylamide Gel Electrophoresis

*F. diplosiphon* strains B481-SD and B481-WT were grown in BG-11 media amended with 0.5, 2.0, and 128 mg/L n-TiO_2_ according to the culture conditions described in Sect. 2.1. Untreated cultures devoid of n-TiO_2_ served as the control. After a cultivation period of 9 days, cells were harvested, total protein extracted and quantified using the BCA assay as described by Gichuki et al. [17]. Samples were diluted to a concentration of 2.0 mg/mL in SDS buffer, heated to 95 °C for 10 min, and run on a 10% sodium dodecyl sulfate (SDS) gel according to the method of Laemmli and modified by O’Farrell [18].

#### Western Blot and Densitometric Analysis

The blots were rinsed in Tween-20 Tris buffer saline (TTBS), destained in 100% methanol, and blocked for two hours in 5% nonfat dry milk (NFDM) in TTBS. After washing for 3 to 10 min in TTBS, the blots were incubated overnight with the primary antibody (anti-ATP synthase beta subunit [Agrisera, Cat# AS05085] diluted 1:50,000 in 2% NFDM TTBS). Band intensities were quantified above background using Phoretix 1D software (version 11.2) on a Windows 10-compatible system. The membranes were treated with secondary anti-Rabbit IgG-HRP antibody (SeraCare, Cat# 5220 − 0337) for 2 h at room temperature as mentioned above. Signal detection was carried out using enhanced chemiluminescence (ECL), and the blots were exposed to Kodak BioMax MR X-ray film (3-minute exposures for KO5401 samples #1–3), and scanned using a laser densitometer (Model PDSI, Molecular Dynamics Inc., Sunnyvale, CA). The scanner was calibrated for linearity using a Neutral Density Filter Set (Melles Griot, Irvine, CA).

### Particle Size Characterization and Charge Distribution of Bare n-TiO₂

The hydrodynamic particle size distribution and surface charge characteristics of n-TiO₂ were determined using dynamic light scattering (DLS) and electrophoretic light scattering (ELS) potential on a Nano Particle Analyzer SZ-100 V2 (HORIBA Scientific, Japan). After dispersion in deionized water, the mixture was sonicated for 10 min to ensure uniformity. DLS measurements were conducted at a fixed scattering angle of 173° obtaining the Z-average hydrodynamic diameter and PDI from intensity-weighted distributions. In addition, zeta potential was quantified using electrophoretic light scattering on the same instrument calibrated according to manufacturer’s specifications. All measurements were performed at 25 °C under standard conditions with an applied electrode voltage of 3.4 V.

### Field Emission Scanning Electron Microscopy (FESEM) Analysis

The surface morphology of the untreated control and n-TiO₂-treated *F. diplosiphon* cultures were examined using a Field Emission Scanning Electron Microscope (FESEM; JSM-7100 F, Model SM-71031SE2A, JEOL Ltd., Japan). Cells were centrifuged, lyophilized, and samples placed in carbon tape and air-dried to remove excess powder. Imaging was performed under high-vacuum conditions at an accelerating voltage of 5–15 kV. Micrographs were acquired at multiple magnifications to evaluate cell morphology, aggregation patterns, and surface structural features.

### Statistical Analysis

Statistical analysis was performed using one-way ANOVA with Tukey’s post hoc test (*p* < 0.05). The fixed-effect ANOVA model, *Yij = µ + αN_i + εij*, where *Y* represents the measured parameter, *µ* overall mean, *αN* is the n-TiO₂ treatment effect, and *εij* is the experimental error, was used. Error bars reflect standard deviation (SD).

## Results

### Impact of n-TiO_2_ Nanoparticles on Growth and Pigment Autofluorescence in *F. diplosiphon* B481-SD and B481-WT

**Fig. 1:**
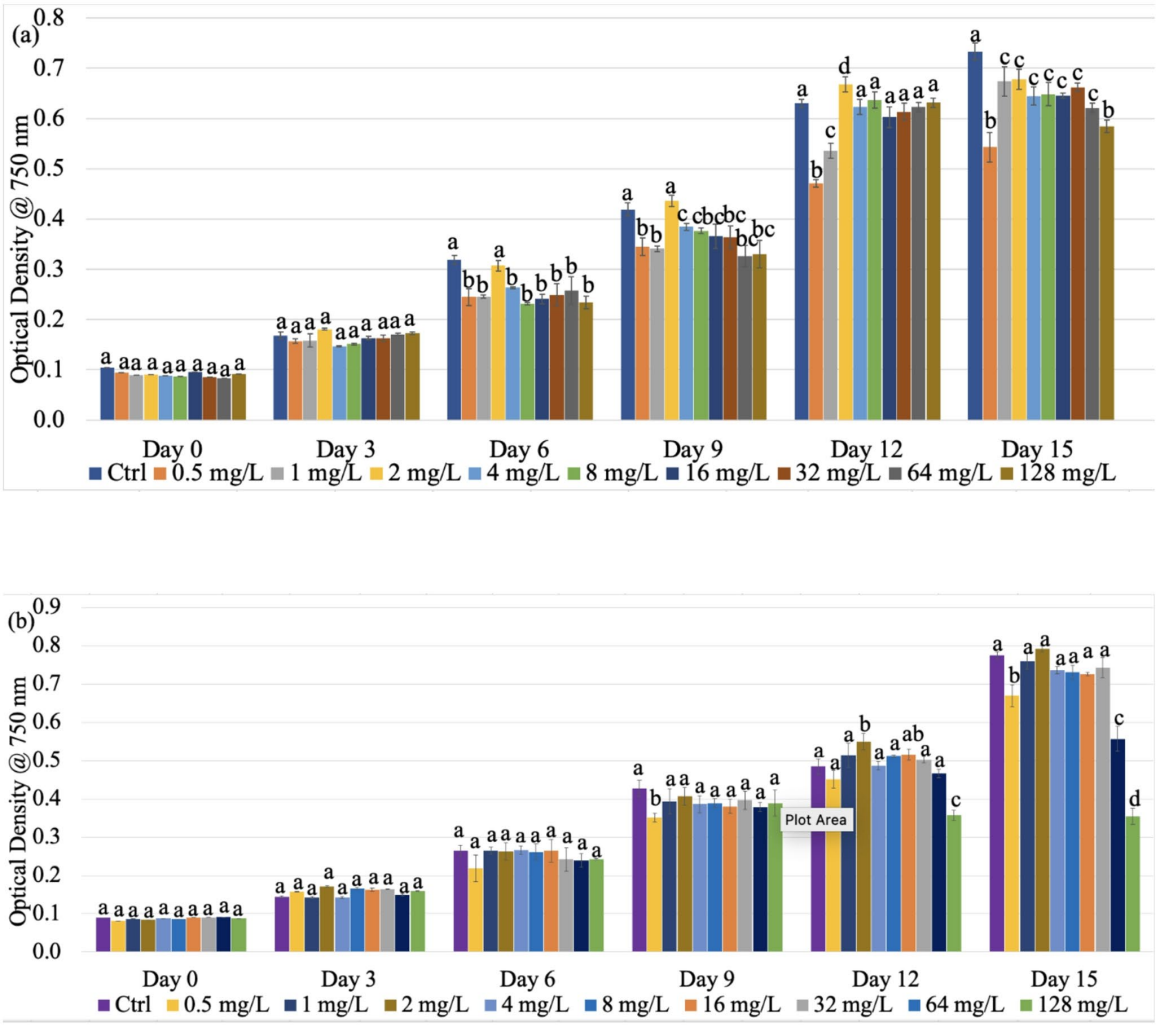
Growth of *Fremyella diplosiphon* (**a**) B481-SD and (**b**) B481-WT strains in nano-titanium dioxide nanoparticles (n-TiO_2_) ranging from 0.5 to 128 mg/L. Different letters above the error bars indicate statistical significance among the various treatments (*p* < 0.05)

Optical density as a measure of cell growth served as an indicator of photosynthesis in *F. diplosiphon* treated with varying n-TiO₂ concentrations. Our results indicated significantly higher growth (*p* < 0.05) of B481-SD at 2.0 mg/L (0.67 ± 0.01) n-TiO_2,_ and at 2.0 (0.55 ± 0.01) and 16 (0.52 ± 0.01) mg/L in B481-WT on day 12 (Fig. 1a, b). Both strains treated with 128 mg/L n-TiO₂ exhibited a significant reduction in growth. Measurement of pigment autofluorescence indicated significantly higher phycocyanin (1300 ± 2) and chlorophyll *a* content (890 ± 5) in B481-SD treated with 2.0 mg/L n-TiO₂ on day 9 compared to the untreated control (Online Resource Figs. 1a and 2a). However, a significant decrease in phycocyanin content was observed on days 12 and 15 in B481-SD treated with 0.5 to 128 mg/L n-TiO_2_. We did not observe significant differences (*p* > 0.05) in phycocyanin or chlorophyll content in B481-WT in 0.5 to 128 mg/L n-TiO_2_ concentrations (Online Resource Figs. 1b and 2b).

**Fig. 2:**
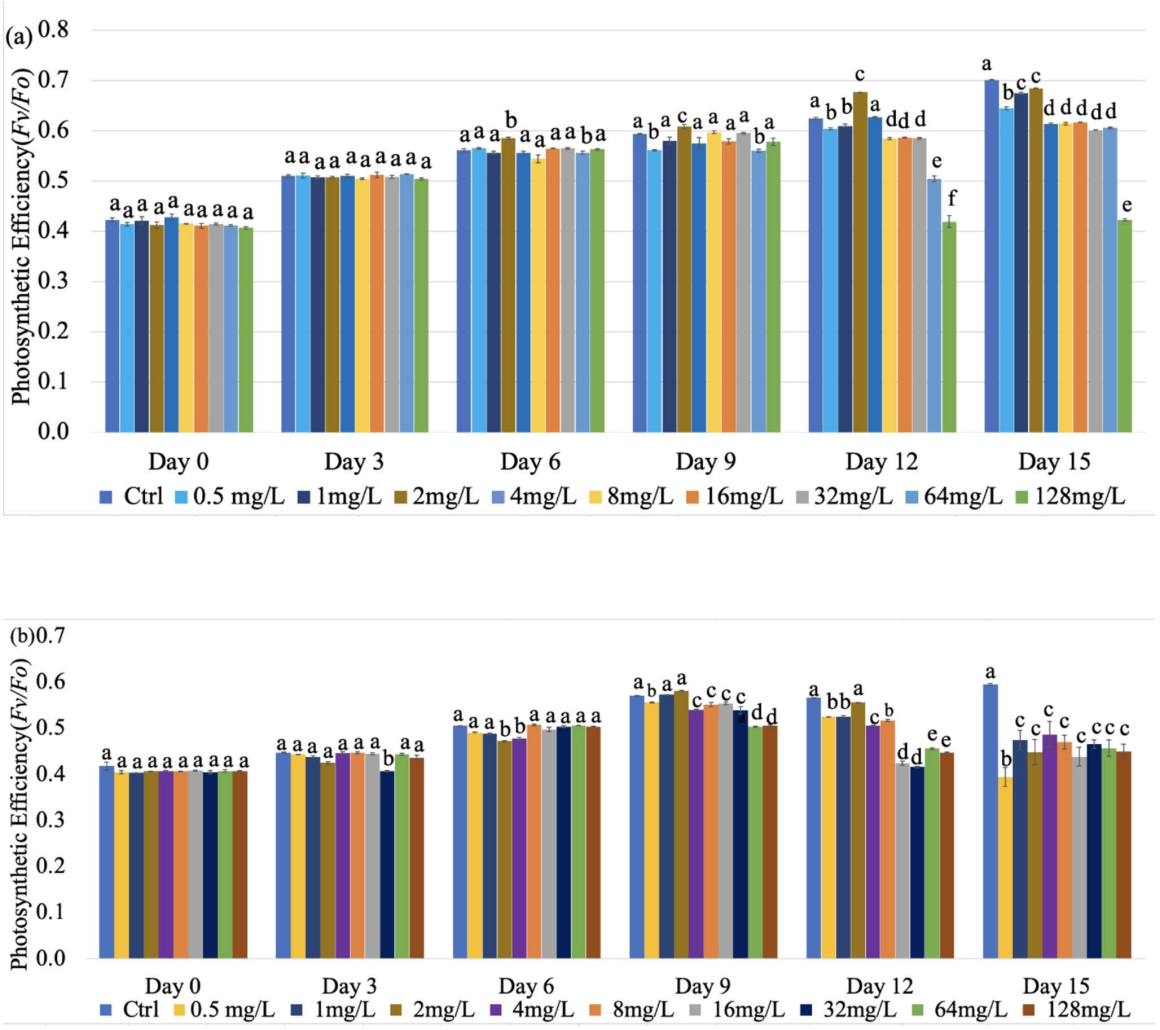
Photosynthetic efficiency (*Fv/Fm)* of *Fremyella diplosiphon* strains (**a**) B481-SD and (**b**) B481-WT treated with nano titanium dioxide nanoparticles (n-TiO_2_) ranging from 0.5 to 128 mg/L. Mean and standard deviations are indicated by error bars. Different letters above the error bars indicate significance among the treatments (*p* < 0.05)

### Photosynthetic Efficacy (*Fv/Fm*) of n-TiO_2_ Treated B481-SD and B481-WT Strains

The quantum yield of PSII was measured to assess the photosynthetic efficiency of n-TiO_2_-treated *F. diplosiphon*. Quantification of the *Fv/Fm* ratio revealed a significant increase (*p* < 0.05) in B481-SD treated with 2.0 mg/L n-TiO_2_ on days 6 (0.58 ± 0.01), 9 (0.61 ± 0.03), and 12 (0.68 ± 0.05) compared to the untreated control (Fig. 2a). On the other hand, we did not observe a significant increase in the *Fv/Fm* ratio in B481-WT across all n-TiO_2_ concentrations ranging from 0.5 to 128 mg/L (Fig. 2b). Both strains exhibited a significant reduction in the *Fv/Fm* ratio at 128 mg/L n-TiO₂ on days 12 and 15, with no signs of recovery (Fig. 2a, b).

### ROS Detection in n-TiO_2_ Treated *F. diplosiphon* via. 2′,7′-Dichlorodihydrofluorescein Diacetate Assay

**Fig. 3:**
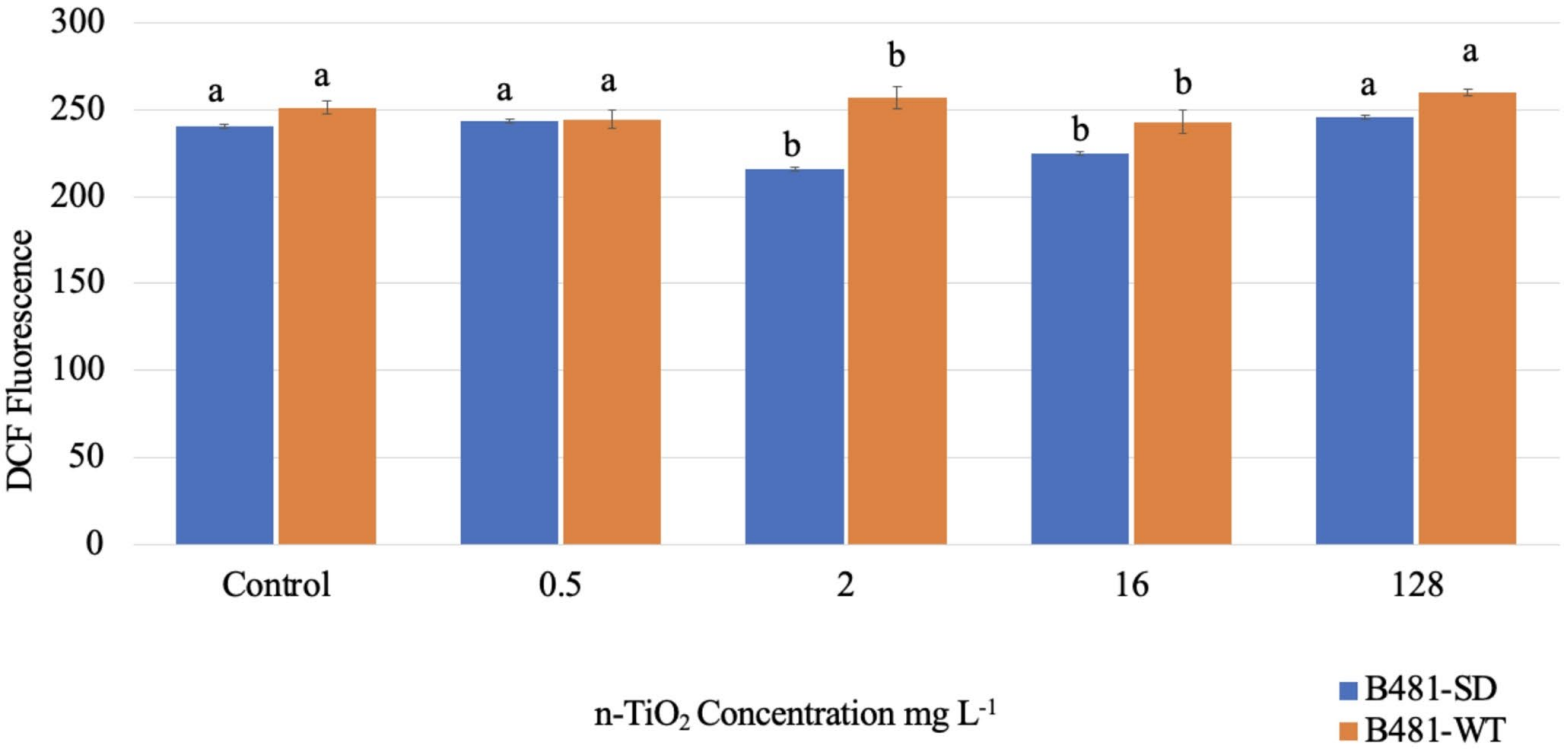
Intracellular reactive oxygen species (ROS) validated using 2′,7′-dichlorofluorescein (DCF) in *Fremyella diplosiphon* B481-SD and B481-WT strains grown in BG-11medium supplemented with 0.5, 2.0, 16 and 128 mg/L nano-titanium dioxide nanoparticles (n-TiO_2_). Average DCF fluorescence (± standard error) of three biological replicates for each sample is shown. Different letters above the error bars indicate significance among treatments

Using the DCFH-DA probe, we compared oxidative stress in the untreated control and at minimal, maximal, and intermediate n-TiO₂ exposure levels (0.5, 2.0, 16, and 128 mg/L) on day 15. While the DCF in B481-SD varied from 220 ± 5 to 245 ± 3 nmol/mL, it ranged from 245 ± 2 to 260 ± 1 nmol/mL in B481-WT. A significant reduction in ROS levels (*p* > 0.01) was observed in B481-SD at 2.0 and 16 mg/L n-TiO₂ compared to the untreated control. Conversely, a significant increase in ROS levels (260 ± 5) was observed in the B481-WT strain exposed to 2.0 mg/L n-TiO₂ (Fig. 3). The negative control with bare nanoparticles showed no interference with the DCF signal.

### Immunodetection of ATP Synthase Levels in n-TiO_2_ Treated *F. diplosiphon*

**Fig. 4:**
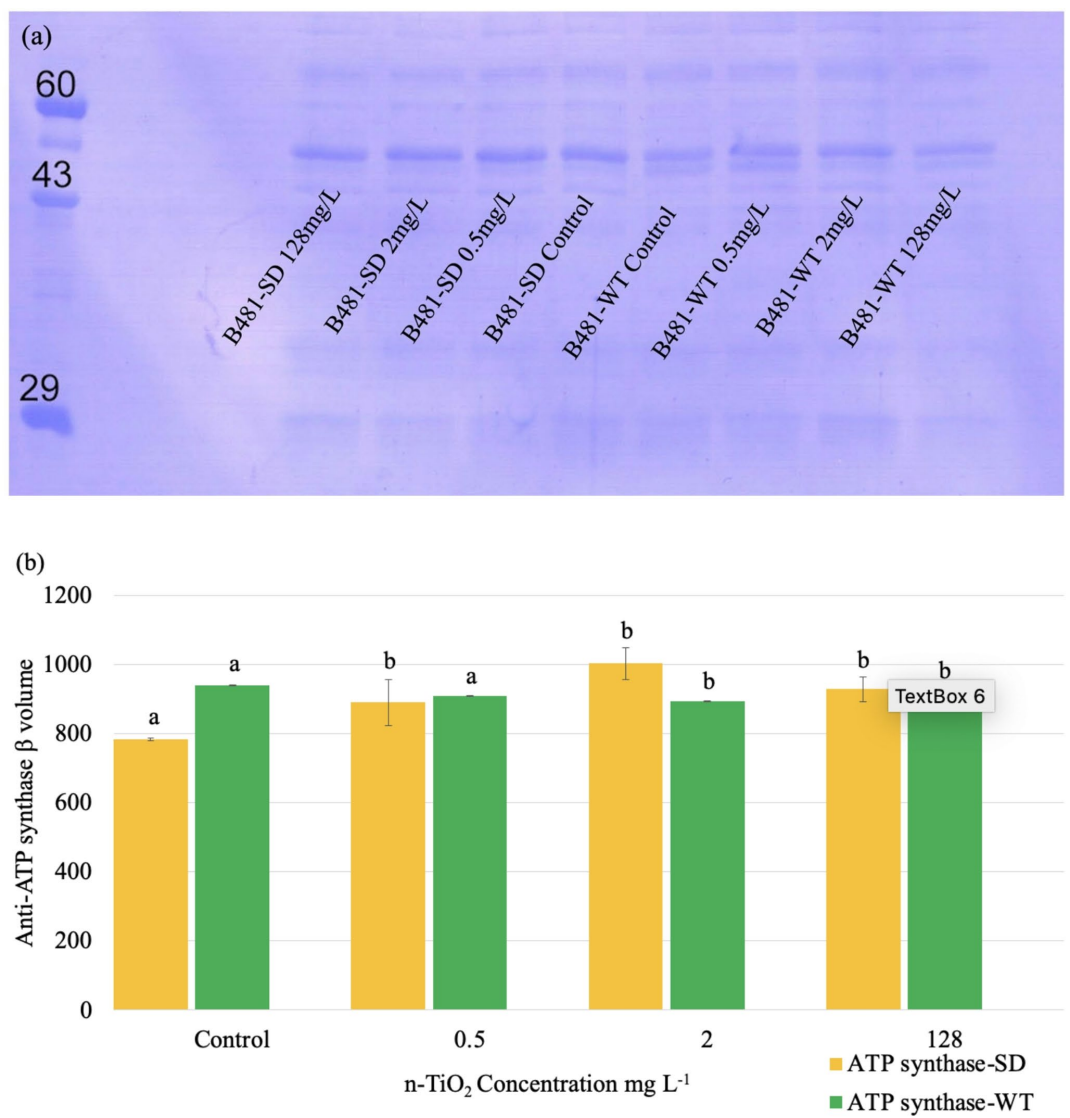
Non-denaturing PAGE and immunoblot analyses of ATP synthase levels in *Fremyella diplosiphon* strains B481-SD and B481-WT grown in BG-11 medium amended with 0.5, 2.0, 16 and 128 mg/L nano-titanium dioxide nanoparticles (n-TiO_2_), (**a**) Immunoblot analysis for detection of ATP synthase protein accumulation, (b) ATP synthase volume in extracted protein lysate. Different letters above the error bars indicate significance among treatments

Densitometric analysis of anti-ATP synthase immunoblots indicated a significant increase in ATP synthase expression in B481-SD treated with 0.5, 2.0, and 128 mg/L n-TiO₂ (*p <* 0.05) compared to the untreated control. The concentrations selected based on preliminary dose–response evaluations were representative of sub-lethal, intermediate, and inhibitory levels. Specifically, we observed significantly higher levels of ATP synthase in B481-SD treated with 2.0 mg/L n-TiO_2_ (1061.666 ± 118.98, *p* < 0.01) compared to 0.5 (997.95 ± 205.20, *p* < 0.01) and 128 mg/L n-TiO_2_ (998.54 ± 130.92, *p* < 0.01). Conversely, no significant increase in ATP synthase expression was observed in B481-WT treated with 0.5, 2.0, or 128 mg/L n-TiO₂ compared to the control. Further, ATP synthase levels were significantly higher in the untreated B481-WT (1017.02 ± 113.67, *p* < 0.05) compared to untreated B481-SD (862.39 ± 138.11, *p* < 0.05). No significant differences in the ATP synthase levels were observed between the two strains at 2.0 or 128 mg/L n-TiO₂ (*p* > 0.05). However, B481-SD exposed to 2.0 mg/L n-TiO₂ exhibited significantly higher ATP synthase activity (1061.67 ± 118.98, *p* < 0.01) compared to B481-WT at the same concentration (961.98 ± 122.58, *p* < 0.01) (Fig. 4).

### Characterization of Particle Size and Charge Distribution of Bare n-TiO₂

DLS analysis of bare n-TiO₂ revealed a hydrodynamic diameter with a Z-average size of 60.2 nm, a mean particle diameter of 56.2 nm, and a modal size of 42.2 nm, indicating a moderately polydisperse distribution (Online Resource Fig. 3a). Zeta potential measurements showed a single peak centered at + 16.6 mV, corresponding to an electrophoretic mobility of 0.000129 cm²/V·s, indicating moderate colloidal stability (Online Resource Fig. 3b). The conductivity of the suspension was 0.195 mS/cm.

### FESEM Imaging and Energy Dispersive X-ray Spectroscopic Analysis

**Fig. 5:**
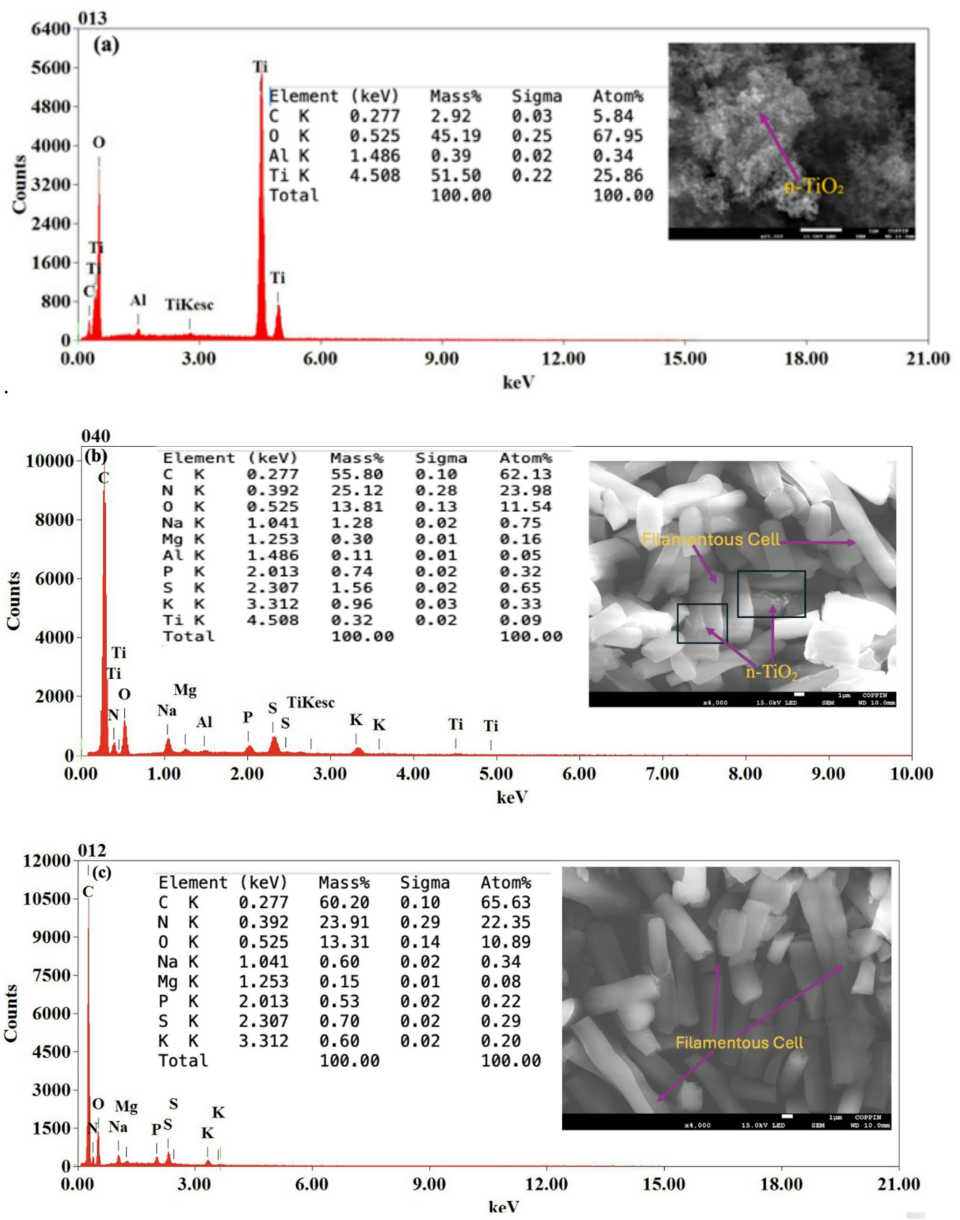
Representative Field Emission Scanning Electron Microscopy (FESEM) images and corresponding EDS showing the atomic percentage of each element in (**a**) pure titanium dioxide nanoparticles (n-TiO₂), (**b**) cultures treated with optimal n-TiO₂, and (**c**) *Fremyella diplosiphon* control culture grown in the absence of n-TiO₂

FESEM equipped with EDS enabled semi-quantitative visualization and analysis of n-TiO₂ distribution in *F. diplosiphon*. The EDS spectrum shown in Fig. 5a represents measurement from a representative region where n-TiO₂ aggregated. While n-TiO₂-treated *F. diplosiphon* exhibited a titanium content of 0.32% as determined by EDS (Fig. 5b), no titanium peak was observed in the spectrum of the untreated control (Fig. 5c). In addition to titanium and oxygen, the spectra indicated the presence of several other elements: including carbon, sodium, sulfur, potassium, magnesium, nitrogen phosphorus, and aluminum, likely originating from the biological matrix of *F. diplosiphon* and residual environmental constituents.

## Discussion

Titanium dioxide nanoparticles have emerged as versatile nanomaterials due to their exceptional photocatalytic, antibacterial, and UV-shielding properties, making them valuable in biotechnological applications [19]. However, at higher concentrations, n-TiO₂ can generate ROS, potentially disrupting cyanobacterial photosynthetic machinery, including photosystem II (PSII) and the electron transport chain [20]. This study established a comparative framework to assess the effects of n-TiO₂ on the physiological performance and metabolic efficiency of *F. diplosiphon*, a cyanobacterium with significant potential for high-value bioproduct and renewable energy generation.

Our findings revealed significantly higher growth of *F. diplosiphon* B481-SD at 2.0 mg/L n-TiO_2_, and B481-WT at 2.0 and 16 mg/L on day 12 (Fig. 1a, b), indicating a hormetic response to nanoparticle exposure. These results are in accordance with the findings of Dedman et al. [21], where a 10% increase in *Prochlorococcus* cell density was observed within 24 h of n-TiO₂ exposure. This increase was attributed to the bio-stimulating effects of nanoparticle degradation products or organic molecules secreted by the cyanobacterium. While BG-11 medium is optimal for cyanobacterial cultivation, it should be noted that it contains 0.001 g/L EDTA and may partially chelate metal-based nanoparticles, reducing their toxicity [22]. Both strains exhibited a significant decrease in growth with no recovery at 128 mg/L n-TiO₂ on day 15 (Fig. 1a, b), indicating potential toxicity that impaired cellular functions (Fig. 1a, b). Similarly, in the green alga *Raphidocelis subcapitata*, a significant decline in growth was reported at 100 mg/L n-TiO₂ [23]. This effect was attributed to excessive oxidative stress from high intracellular nanoparticle accumulation, causing extensive damage to cellular components and disrupting essential processes.

To assess the impact on photosynthesis, we quantified pigment accumulation in B481-SD and B481-WT strains treated with 0.5 to 128 mg/L n-TiO_2_. A significant increase in phycocyanin and chlorophyll content was observed in B481-SD at 2.0 mg/L n-TiO_2_ (Online Resource Figs. 1a and 2a), suggesting the activation of cellular signaling pathways triggered by these nanoparticles. Similar findings were reported in *Synechococcus cedrorum*, where enhanced pigment accumulation associated with defense and tolerance responses was reported at 25 mg/L n-TiO_2_ after 15 and 30 min of exposure [24]. Similarly, exposure of *Scenedesmus obliquus* to 10, 50, and 100 nm-sized n-TiO₂ resulted in a 55% increase in Chl *b*/Chl *a* ratio compared to the control [11]. The observed reduction in phycocyanin content in *F. diplosiphon* B481-SD upon exposure to 0.5–128 mg/L n-TiO₂ on days 12 and 15 (Online Resource Fig. 1a) is likely attributed to nanoparticle-induced cytotoxicity, including oxidative stress and disruption of metabolic pathways essential for phycobiliprotein synthesis. These findings are consistent with those of Boutarfa et al. [25], who reported a significant decrease in phycocyanin levels in *Microcystis* sp. after exposure to 600 mg/L n-TiO₂. Similarly, Yalcin et al. [13] demonstrated a marked inhibition of phycocyanin production in *F. diplosiphon* B481-SD when treated with 51.2 mg/L nanoscale zero-valent iron nanoparticles (nZVIs), further supporting the sensitivity of cyanobacterial pigment synthesis pathways to nanoparticle-mediated stress. In contrast, no significant increase in phycocyanin or chlorophyll content was detected in the wild-type strain across all tested n-TiO₂ concentrations (0.5–128 mg/L) (Online Resource Figs. 1b and 2b), suggesting potential strain-specific differences in pigment regulation and nanoparticle interaction mechanisms. The reduction in pigment levels may be attributed to the disruption of photosynthetic pigment biosynthesis and impairment of the photosynthetic electron transport chain, resulting in diminished photosystem II (PSII) activity and overall photosynthetic efficiency [26].

Photosynthetic capacity as a measure of the *Fv/Fm* ratio revealed a significant enhancement in B481-SD at 2.0 mg/L n-TiO₂ (Fig. 2a), suggesting possible activation of antioxidant defense systems or structural modifications in the photosynthetic apparatus to enhance light harvesting and energy utilization. This observation aligns with findings in *Chlamydomonas reinhardtii*, where an increased *Fv/Fm* ratio was reported following exposure to 200 mg/L n-TiO₂, likely as a response to excessive absorbed light energy surpassing the capacity for photochemical use, thereby inducing protective mechanisms [27]. Although cyanobacteria generally exhibit relatively lower maximum quantum yield values in the range of 0.2–0.5, the values observed in this study (0.4–0.6) are consistent with our previous findings in *F. diplosiphon* strains B481-WT and B481-SD, where *Fv/Fm* values ranged from 0.4 to 0.8 in the control and ampicillin-treated cells [28]. It is possible that variations in PSII organization and physiological responses may account for these differences. Notably, the sterol desaturase mutant showed improved growth and higher *Fv/Fm* values under n-TiO₂ exposure, likely attributable to enhanced ROS scavenging potential. In contrast, at 128 mg/L n-TiO₂, both B481-SD and B481-WT strains exhibited a significant reduction in *Fv/Fm* values on days 12 and 15 (Fig. 2a and b), indicating substantial impairment of photosystem II (PSII) function under high nanoparticle stress. Notably, B481-WT treated with n-TiO₂ did not exhibit a significant increase in *Fv/Fm* ratio (Fig. 2b), further supporting the existence of physiological or regulatory differences between the strains in their responses to nanoparticle exposure. A comparable decline in photosynthetic efficiency was reported in *Synechocystis* sp. PCC 6803, where photocurrent generation decreased at 10 mg/L n-TiO₂ due to nanoparticle-induced shading effects that limited light availability and hindered photosynthetic performance [29].

ROS levels were significantly elevated in B481-WT at 2.0 and 128 mg/L n-TiO₂ (Fig. 3), likely due to nanoparticle-induced cell membrane disruption, interference with energy transduction, and reduced light availability due to nanoparticle aggregation. In a study by Liu et al. [27], increased production of ROS and subsequent disruption of redox homeostasis were reported in *Chlamydomonas reinhardtii* at 20 mg/L n-TiO_2_. In contrast, the B481-SD strain exhibited significantly reduced ROS levels at 2.0 and 16 mg/L n-TiO₂ (Fig. 3), indicating enhanced antioxidant defense responses compared to B481-WT. This strain-specific difference in oxidative stress tolerance may reflect distinct regulatory mechanisms that restrain ROS detoxification. A comparable reduction in ROS content was reported in *Skeletonema costatum* following n-TiO₂ exposure, attributed to inhibited electron transport in chloroplasts, which limits ROS generation [30]. These findings are further supported by Gichuki et al. [17], who observed lower autofluorescence in B481-SD relative to B481-WT across nZVI concentrations of 3.2 to 25.6 mg/L, reinforcing the notion of differential ROS modulation between strains under nanoparticle-induced stress. The interaction of n-TiO₂ with cyanobacteria induces electron transfer in metabolic reactions by facilitating the production of oxygen and hydroxyl radicals through electron release, causing a toxic response in aquatic organisms and a decrease in ROS levels [31].

Photosynthetic protein complexes, including photosystem I, cytochrome b6f, and photosystem II, are integral to energy generation and storage in prokaryotes, facilitating essential processes required for cellular metabolism [32]. In our study, ATP synthase protein levels were significantly elevated in B481-SD at 0.5, 2, and 128 mg/L n-TiO₂ concentrations compared to the untreated control (Fig. 4a, b), indicating a cellular adaptation mechanism to nanoparticle-induced stress involving enhanced photosynthetic recovery. The observed effect could be attributed to altered metabolic signaling affecting gene expression pathways involved in energy metabolism in B481-SD. A previous transcriptome analysis of B481-SD treated with 3.2 mg/L nZVIs revealed a log2 fold increase ranging from 1.1X to 1.7X due to the upregulation of photosynthetic genes such as *psaI, psaX, psaM, psbK, psbA*, and *psb30* compared to the untreated control [33]. In addition, the resilience of the strain to the combination regimen of 0.8 mg/L ampicillin and 3.2 mg/L nZVIs (*p < 0.05*) suggests a strain-specific regulatory network and stress responses [34]. The significantly enhanced growth, pigment autofluorescence, ATP synthase activity, and higher *Fv/Fm* values in n-TiO₂-treated B481-SD could be attributed to the sterol desaturase gene, which is known to increase cyanobacterial membrane fluidity and stability under stress conditions [35]. Enhanced membrane fluidity can facilitate more efficient electron transport across thylakoid membranes providing protection against nanoparticle-induced oxidative stress, thereby reducing membrane damage [36]. These adaptations or protective mechanisms could have contributed to improved photosynthetic performance and the maintenance of physiological homeostasis in the engineered strain, resulting in tolerance to n-TiO₂ exposure.

DLS analysis indicated substantial aggregation of n-TiO₂ in aqueous suspension, with a Z-average size of 60.2 nm, nearly threefold higher than the nominal particle size of 20 nm. This size increase may be attributed to nanoparticle agglomeration, hydration shell formation, and the intensity-weighted nature of DLS measurements. Similar findings were reported by Oikawa et al. [37], who observed average particle sizes of 50 ± 150 nm, underscoring the variability associated with nanoparticle dispersion. We observed a zeta potential of + 16.6 mV, which was within the ± 10 to ± 30 mV range typically associated with incipient colloidal stability with a tendency toward aggregation due to surface hydroxyl group protonation as reported by Chuc et al. [38]. No additional peaks were detected, confirming a single dominant nanoparticle population despite the broad size distribution.

FESEM imaging confirmed aggregation of n-TiO₂ and its close association with *F. diplosiphon* filaments, likely through surface adsorption. EDS analysis further validated the presence of titanium in treated cultures (0.32% atomic percentage), while the untreated control did not exhibit detectable titanium signal, validating the uptake or surface binding of nanoparticles in n-TiO₂-treated cultures. Although EDS cannot resolve internal versus surface-bound nanoparticles, the findings suggest successful interaction between n-TiO₂ and the cyanobacterial cells. These interactions may influence cell surface properties, light scattering, and physiological responses [39]. Our results are consistent with prior observations in *Prochlorococcus*, where significant physiological and biochemical effects were induced even at 0.1% n-TiO₂ incorporation [21]. The detection of additional elements such as carbon, oxygen, sodium, and phosphorus reflect the native composition of the biomass and medium-derived mineral content.

## Conclusion

This study highlights the differential physiological responses of wild-type and sterol desaturase-overexpressing *F. diplosiphon* strains to n-TiO₂ nanoparticle exposure. The mutant strain exhibited enhanced n-TiO₂ tolerance, suggesting the protective role of altered membrane lipids against nanoparticle-induced stress. These findings provide new insights into the interaction between cyanobacterial membrane dynamics and nanomaterials, with potential applications in developing stress-resilient cultivation systems. Future investigations will focus on transcriptome analysis to elucidate the regulatory dynamics of gene expression associated with n-TiO₂ uptake to advance the development of large-scale bioproducts from *F. diplosiphon* strain B481-SD. Future investigations will focus on transcriptome analysis to elucidate the regulatory dynamics of gene expression associated with n-TiO₂ uptake and to advance the development of large-scale bioproducts from *F. diplosiphon* strain B481-SD.

## Supplementary Information

Below is the link to the electronic supplementary material.

Supplementary Material 1

## Data Availability

Data is contained within the article.
