# Supplementary material for: Strain-Specific Impact of Titanium Dioxide Nanoparticles on Fremyella Diplosiphon Physiological and Metabolic Responses
Source: Curr Microbiol. 2025 Dec 1;83(1):53. doi: 10.1007/s00284-025-04629-9 (PMC12665634; doi:10.1007/s00284-025-04629-9)
Supplement: Supplementary file 1 — Supplementary Material 1 [file 284_2025_4629_MOESM1_ESM.docx]

**Strain-Specific Impact of Titanium Dioxide Nanoparticles on *Fremyella diplosiphon* Physiological and Metabolic Responses**

Mst Sayadujjhara, Yavuz S. Yalcin, William Ghann, Jamal Uddin, and Viji Sitther. Department of Biology, Morgan State University, Baltimore, MD 21251, USA; Center for Nanotechnology, Department of Natural Sciences, Coppin State University, 2500 West North Avenue, Baltimore, Maryland 21216, USA

**Corresponding Author**: Viji Sitther - https://orcid.org/0000-0003-0096-569X

Email address: viji.sitther@morgan.edu

**Electronic Supplementary Material**

Fig. 1. Phycocyanin autofluorescence of *Fremyella diplosiphon* strains (a) B481-SD and (b) B481-WT measured at a 590 nm excitation and 650 nm emission. Both strains were grown in nano-titanium dioxide nanoparticles (n-TiO_2_) ranging from 0.5 to 128 mg/L. Different letters above the error bars indicate signiﬁcance among treatments (*p* < 0.05).

Fig. 2. Chlorophyll *a* autofluorescence of *Fremyella diplosiphon* strains (a) B481-SD and (b) B481-WT measured at an excitation of 420 nm and emission of 680 nm. Both strains were grown in nano-titanium dioxide nanoparticles (n-TiO_2_) ranging from 0.5 to 128 mg/L. Different letters above the error bars indicate signiﬁcance among treatments (*p* < 0.05).

Fig. 3. Size and surface charge distribution of bare titanium dioxide nanoparticles (n-TiO₂) in aqueous solution, measured by dynamic light scattering. (a) Frequency (%) versus diameter (nm) curve showing a Gaussian-like particle size distribution; the mean (red dashed line), mode (green dotted line), and Z-average size (purple dash-dotted line) are denoted. (b) Zeta potential distribution of n-TiO₂ nanoparticles displaying a single, unimodal peak.


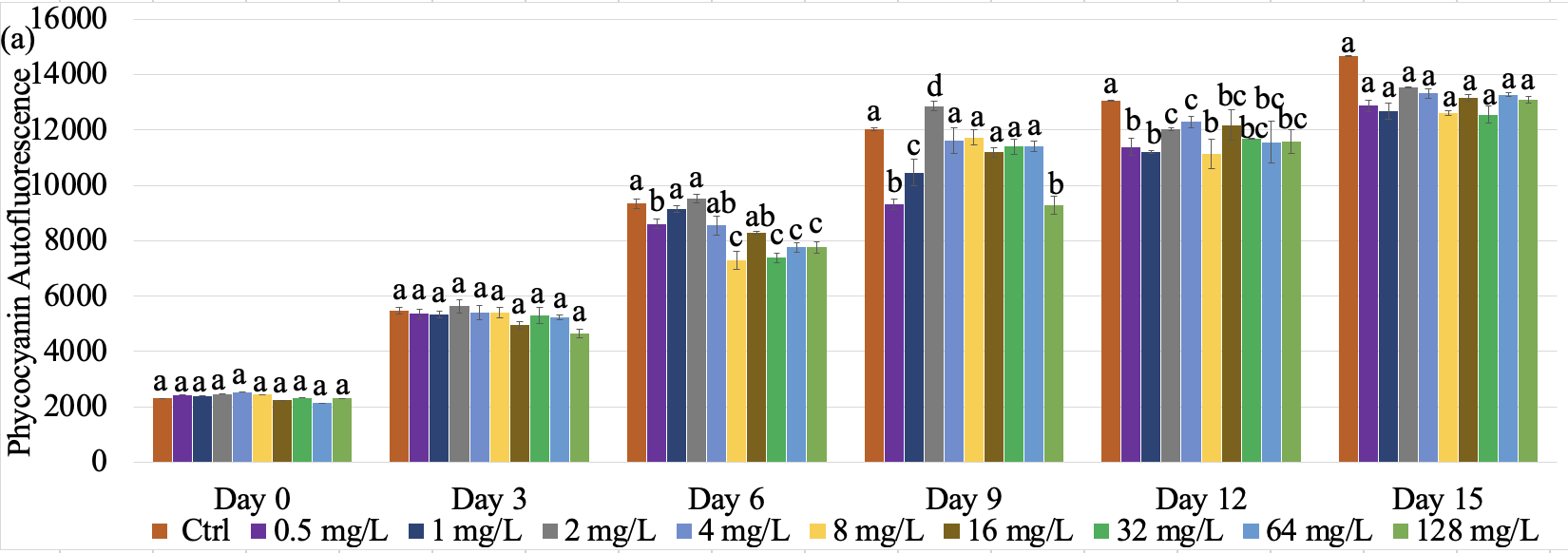


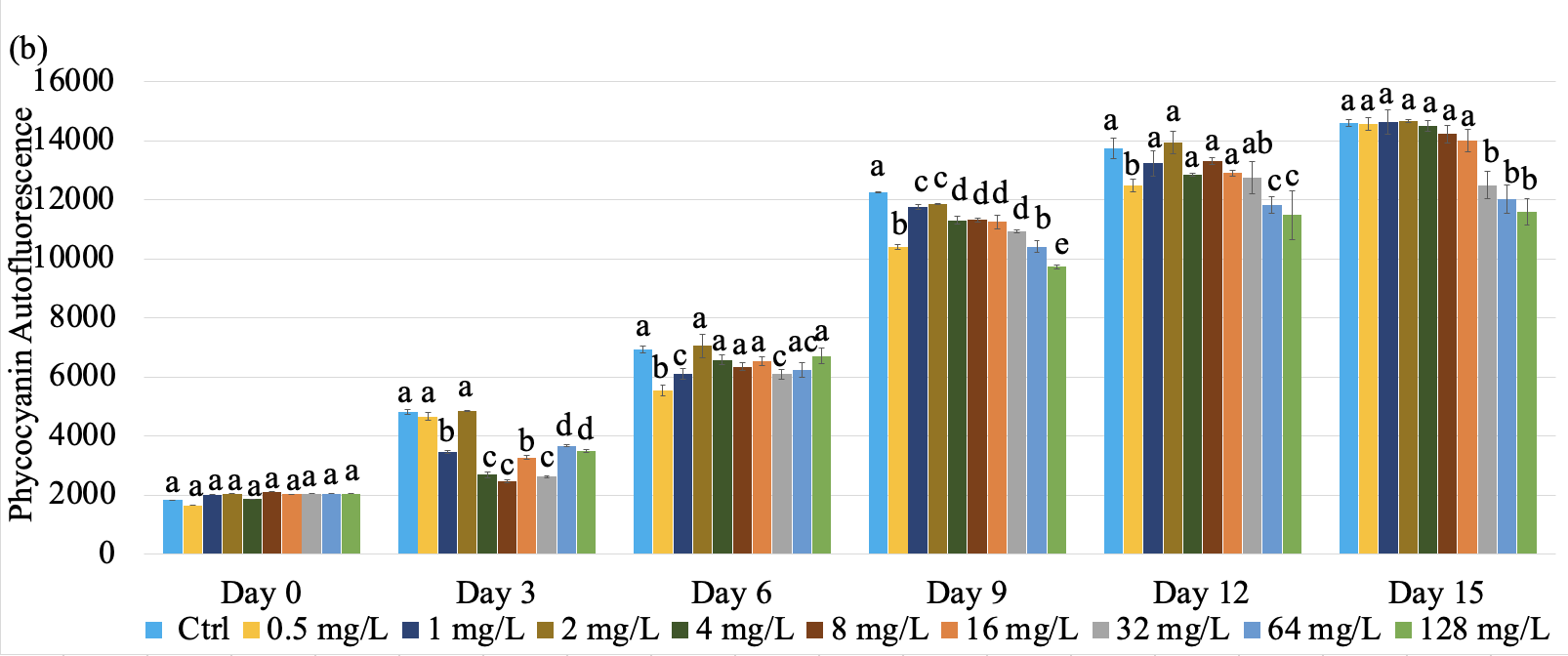


Fig. 1. Phycocyanin autoﬂuorescense of *Fremyella diplosiphon* strains (a) B481-SD and (b) B481-WT measured at a 590 nm excitation and 650 nm emission. Both strains were grown in nano-titanium dioxide nanoparticles (n-TiO_2_) ranging from 0.5 to 128 mg/L. Different letters above the error bars indicate signiﬁcance among treatments (*p* < 0.05).


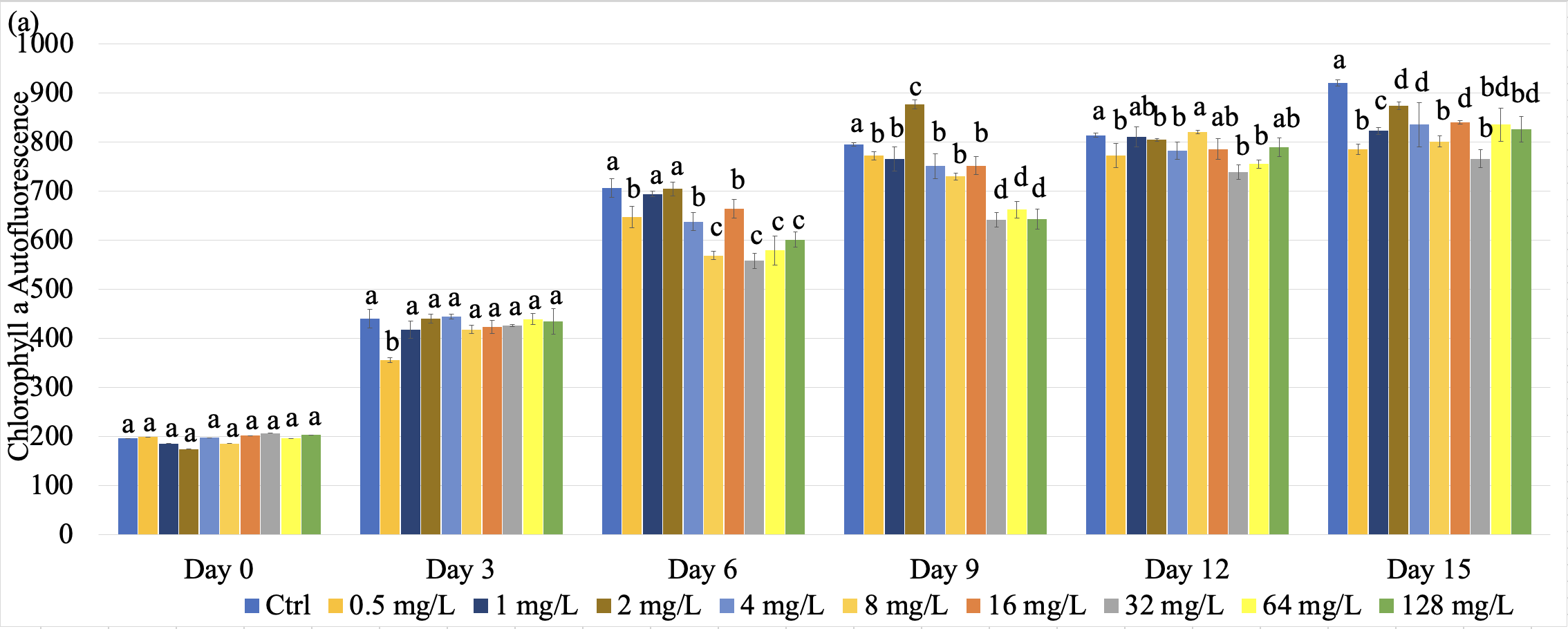


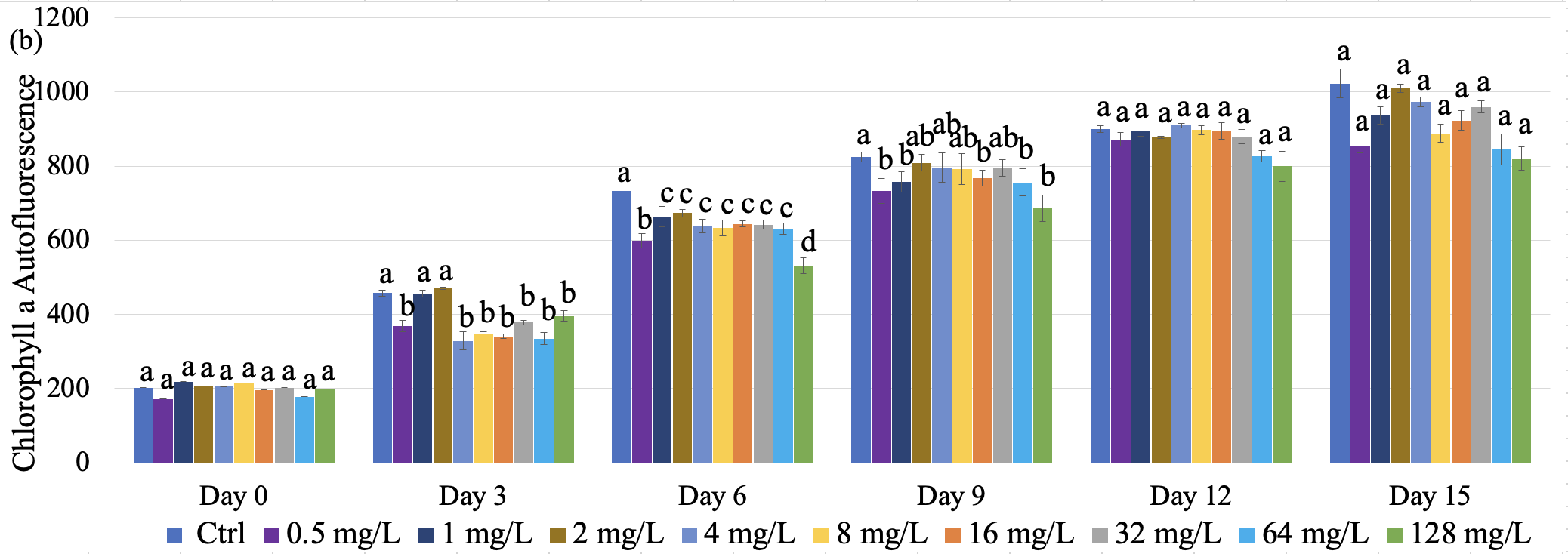


Fig. 2. Chlorophyll *a* autofluorescence of *Fremyella diplosiphon* strains (a) B481-SD and (b) B481-WT measured at an excitation of 420 nm and emission of 680 nm. Both strains were grown in nano-titanium dioxide nanoparticles (n-TiO_2_) ranging from 0.5 to 128 mg/L. Different letters above the error bars indicate signiﬁcance among treatments (*p* < 0.05).


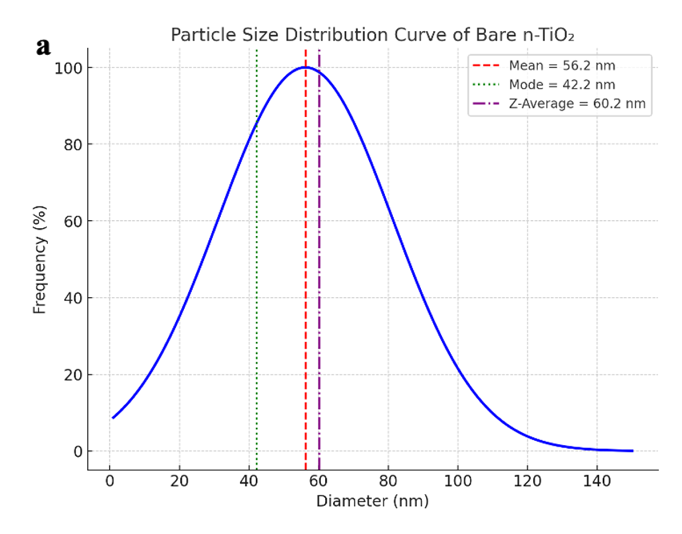

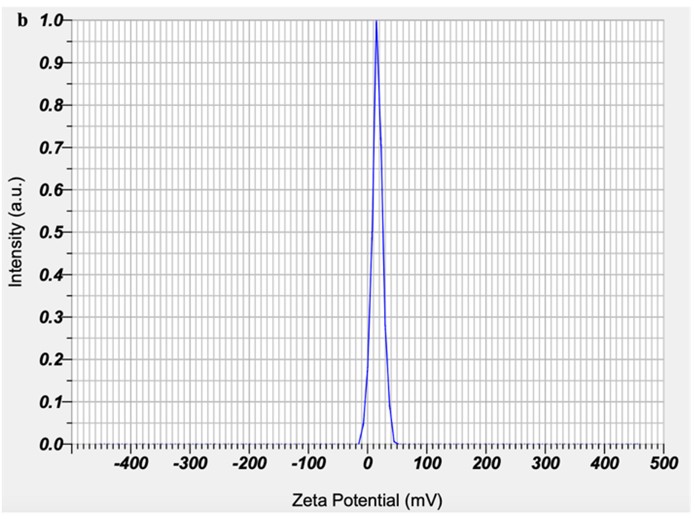


Fig. 3. Size and surface charge distribution of bare titanium dioxide nanoparticles (n-TiO₂) in aqueous solution, measured by dynamic light scattering. (a) Frequency (%) versus diameter (nm) curve showing a Gaussian-like particle size distribution; the mean (red dashed line), mode (green dotted line), and Z-average size (purple dash-dotted line) are denoted. (b) Zeta potential distribution of n-TiO₂ nanoparticles displaying a single, unimodal peak.

.
